# Supplementary material for: microRNA Targeting Cytochrome P450 Is Involved in Chlorfenapyr Tolerance in the Silkworm, Bombyx mori (Lepidoptera: Bombycidae)
Source: Insects. 2025 May 12;16(5):515. doi: 10.3390/insects16050515 (PMC12112709; doi:10.3390/insects16050515)
Supplement: Supplementary file 1 [file insects-16-00515-s001.zip › Supplementary Figures.pdf]

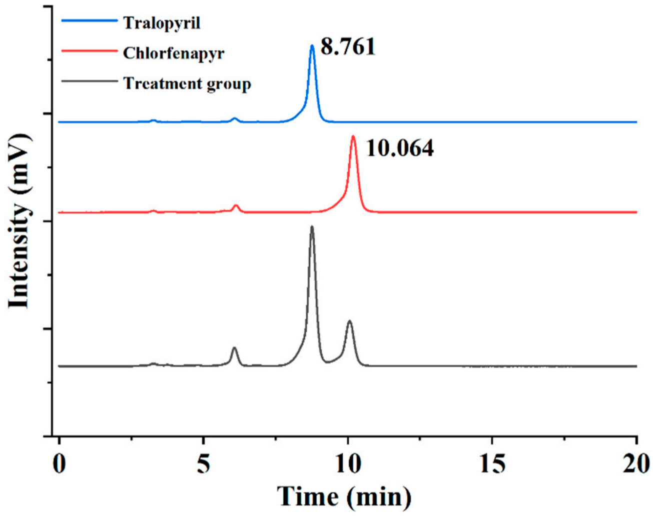

Figure S1. Chromatofram of standard chlorfenapyr and tralopyril showing the retention time of these two chemicals.

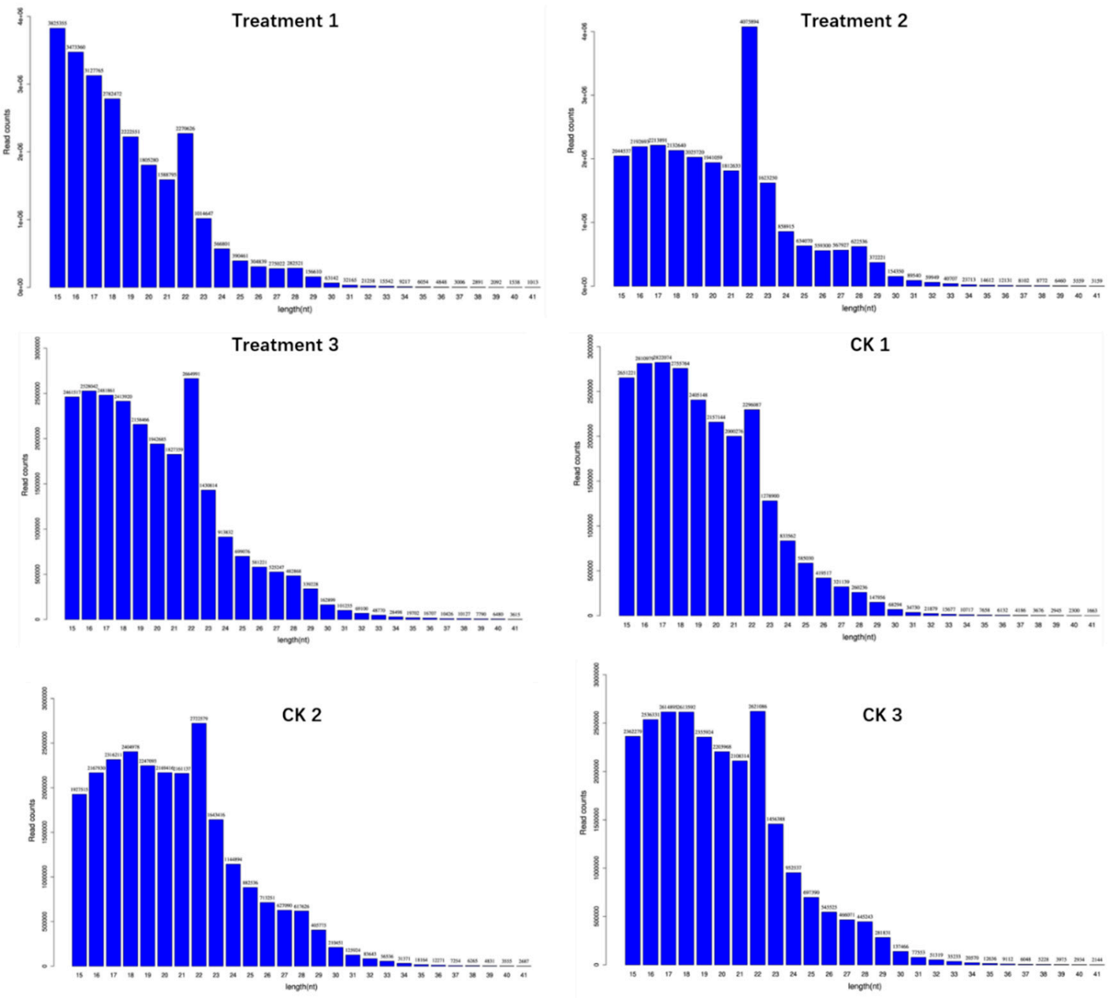

Figure S2. Total small RNA length distribution of the chlorfenapyr treated *B. mori*

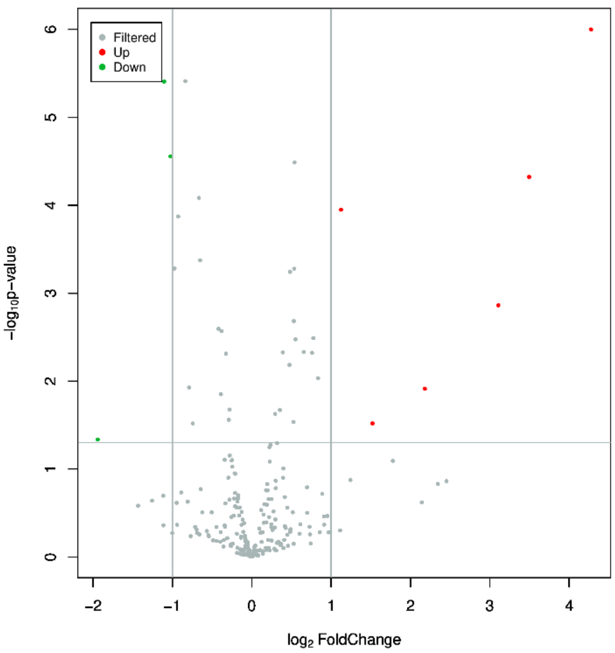

Figure S3. The volcano figure of Differential expressed miRNAs between chlorfenapyr treatment and healthy *B. mori* larvae. Gray dots represent the non-differentially expressed miRNAs, red dots (up-regulated) and green dots (down-regulated) represent the differentially expressed miRNAs

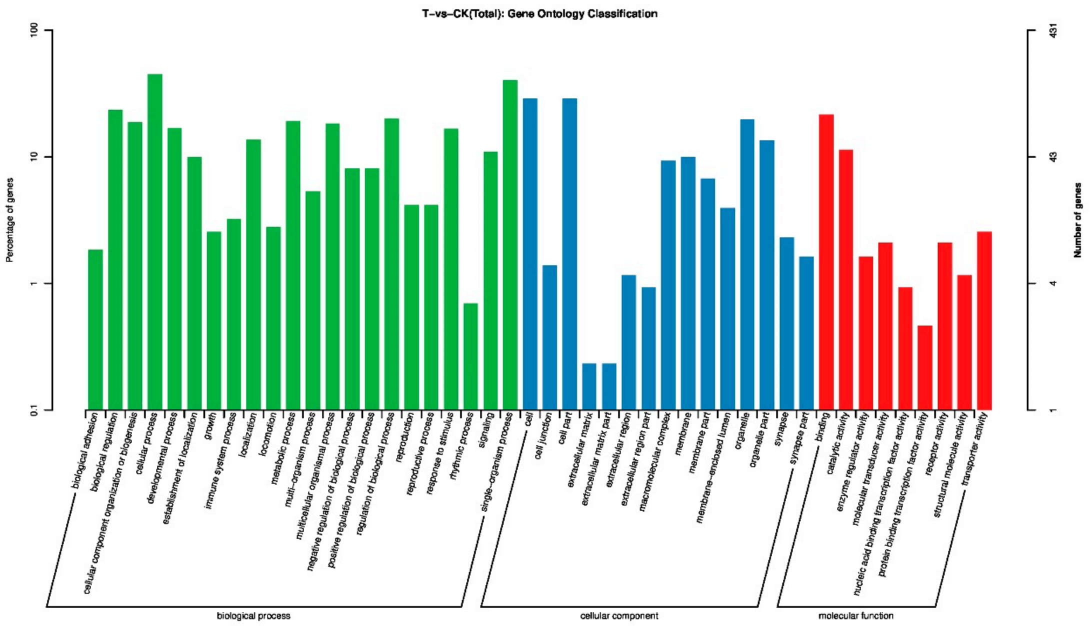

Figure S4. Functional categories of the differentially expressed target genes in the GO database.

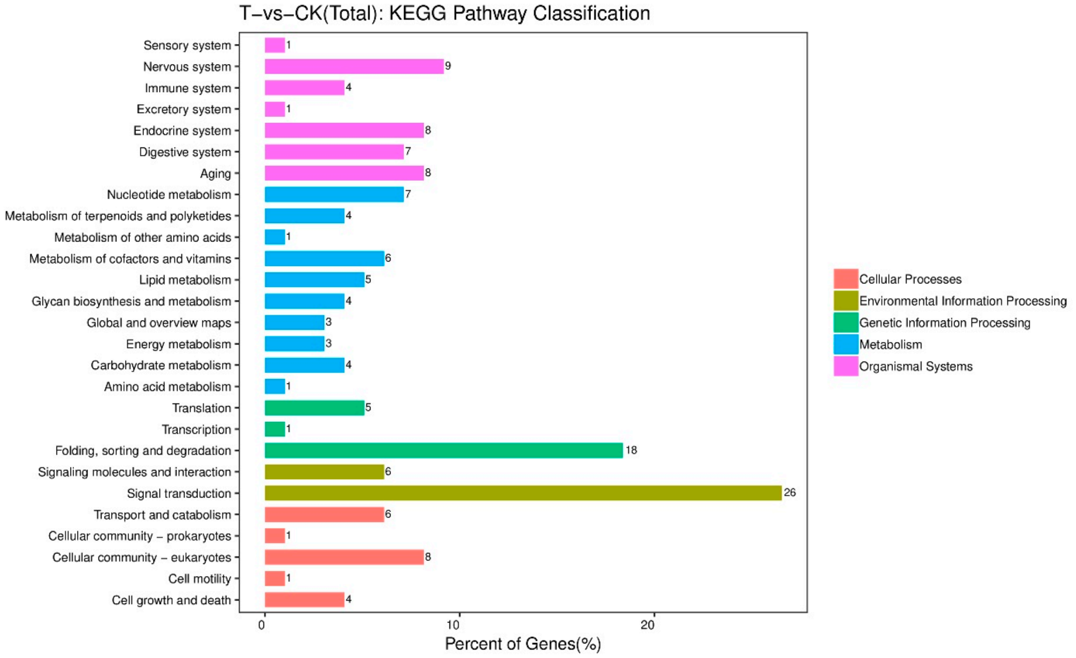

Figure S5. Functional categories of target genes of differentially expressed miRNAs in KEGG pathways. These genes were significantly enriched in 27 subpathways under five main pathways.

CYP337A2 / bmo-miR-6497-5p predicted interation

```
mfe: -35.7 kcal/mol
target 5'     G  G             A 3'
               GC CUGGUCCUCAGAG
               CG GGCCAGGAGUCUC
miRNA  3' CUGUG  G             G 5'
```

CYP6AE3P / bmo-miR-6497-5p predicted interation

```
mfe: -31.1 kcal/mol
target 5' U    C A    A   GA       A 3'
           GCGC G CCCC GGU  CUCGGAG
           UGUG C GGGG CCA  GAGUCUC
miRNA  3' C               G        G 5'
```

CYP49A1 / bmo-miR-6497-5p predicted interation

```
mfe: -25.5 kcal/mol
target 5' G       A      GGA       U 3'
           GACAC C   GGUU   UCGGAGU
           CUGUG G   CCAG   AGUCUCG
miRNA  3'      C GGG    G            5'
```

CYP333B1 / bmo-miR-6497-5p predicted interation

```
mfe: -28.6 kcal/mol
target 5' C      G  AUUCG        A 3'
           GCGCGC UC     UCUCAGAG
           UGUGCG GG     GGAGUCUC
miRNA  3' C      G  CCA          G 5
```

Figure S6. Prediction of target gene binding regions between Bmo-miR-6497-5P and P450s.
