# Supplementary material for: microRNA Targeting Cytochrome P450 Is Involved in Chlorfenapyr Tolerance in the Silkworm, Bombyx mori (Lepidoptera: Bombycidae)
Source: Insects. 2025 May 12;16(5):515. doi: 10.3390/insects16050515 (PMC12112709; doi:10.3390/insects16050515)
Supplement: Supplementary file 1 [file insects-16-00515-s001.zip › Supplementary Tables.pdf]

Table S1 Primers used for real-time qRT-PCR

Gene names	Sequence (5'-3')
BmCYP333B1F	CTTCAAACACGATGTCCGCA
BmCYP333B1R	ATGGCCTCTTTGATGCAAGC
BmCYP6AE3PF	CTTTCCTGACCCGGAAGAGT
BmCYP6AE3PR	ATTTTCCCGAAGCGCATACC
BmCYP49A1F	AGGAGACTTGAGGAACTGCG
BmCYP49A1R	CTCCTGATTTTGTCCGAGCG
BmCYP337A2F	GTTGGCAAGCAACTTCCAGA
BmCYP337A2R	CCAACGAGGCAGATCGTCTA
BmU6F	CCTGCGCAAGGATGAC
BmU6R	GTGCAGGGTCCGAGGT
Bmo-miR-6497-5p F	GCTCTGAGGACCGGGGCGTGTC
Bmo-miR-6498-3p	AACGTCTGCGATGATACAGTT
Bmo-miR-6498-5p	CGCGTCTGTTGTCGCAGCCGTGC
Bmo-miR-2999	CTGCGACGGACTAGACGCGCA
U6F	TGCGCAAGGATGACACGCAAAATC

Table S2 Sequencing raw data and its filtering

Samples	Raw reads	Reads trimmed length	Reads trimmed Q20	Reads trimmed N	Clean reads	Clean reads uniq
Chlorfenapyr treatement1	40.44M	24.39M	24.29M	24.25M	24.25M	1.76M
Chlorfenapyr treatment 2	31.19M	24.21M	24.15M	24.10M	24.10M	1.87M
Chlorfenapyr treatment 3	33.47M	24.06M	23.98M	23.94M	23.94M	1.99M
Control group 1	32.10M	24.05M	23.97M	23.92M	23.92M	1.72M
Control group 2	30.08M	24.83M	24.76M	24.71M	24.71M	1.95M
Control group 3	31.61M	24.75M	24.67M	24.63M	24.63M	1.93M

Table S3 miRNA categories and their percentage of total reads in each sample of *B. mori*

Samples	rRNA	tRNA	snRNA	Cis-reg	Others
Chlorfenapyr treatement 1	14,424 (1.20%)	3,549 (0.51%)	19,058 (1.36%)	16,186 (1.23%)	28,193 (1.78%)
Chlorfenapyr treatement 2	10,543 (0.76%)	2,623 (0.37%)	14,136 (0.79%)	12,083 (0.78%)	20,617 (1.17%)
Chlorfenapyr treatement 3	11,844 (0.73%)	2,998 (0.42%)	15,472 (0.84%)	13,315 (0.87%)	23,086 (1.25%)
Control group 1	12,574 (0.94%)	2,857 (0.32%)	15,419 (0.88%)	13438 (0.94%)	22638 (1.54%)
Control group 2	10,463 (0.69%)	2,446 (0.25%)	12,634 (0.69%)	11,084 (0.62%)	18,856 (1.07%)
Control group 3	11,882 (0.83%)	2,786 (0.27%)	14,659 (0.82%)	12,746 (0.79%)	21,886 (1.27%)

Table S4 Novel miRNAs and their consensus mature sequences in sRNA sequencing

Novel id	Consensus mature sequence	Novel id	Consensus mature sequence
novel1	aacacaccugaaaguuugcucac	novel43	agcgggcgcacgcgucgcu
novel2	uacagcugguugaaggggaccaa	novel44	agagccgcgugugucuuaugau
novel3	auacgcgggaugagauguccuacu	novel45	accgacucagugguguagauua
novel4	aguagacucuaccccaugaucugua	novel46	aguaacagcucagcaccagau
novel5	cagcuguccaaaauauaaaucuga	novel47	cauggucucaucauucaca
novel6	aaagcggaacacgauuacgca	novel48	acgaaagcccggaucuacagggu
novel7	gguggaucuugaugauuucgauuua	novel49	caacugucauuaauuuuaaaa
novel8	acccagauaaguaggacuaacua	novel50	ugugccggaugaugaaacguuu
novel9	agggacggucacguuaugaa	novel51	caaccaagaauaucaaacauaucu
novel10	gugagagcgggggcacaugacg	novel52	uagucuugaacuuguggu
novel11	gaucauacguaccgaguaaacggg	novel53	ugcgucgguugagucggucgagu
novel12	agggacggucacguuaugaa	novel54	ccgugaauucuuccagugccauu
novel13	agaaucgaacucgggacc	novel55	cgugaccacagacauguau
novel14	ucgcucacucaaggagguugug	novel56	cgugaccacagacaugua
novel15	aacaacaaaaucacuagucuucca	novel57	caccauccuaccuauuuga
novel16	aaagggaaggucuuccaccgag	novel58	cacaaccagcuaaccacacugcca
novel17	acauaccauggucucgcaucuguua	novel59	gaugucuggaaccuucuauugcg
novel18	ccccauagccagaacgauggcaaa	novel60	gaugucuggaaccuucuauugcg
novel19	uagcagugcuuuugaucgaguu	novel61	cgacggaucggugcggaaag
novel20	ucaaguacucggagaaccca	novel62	gaugucuggaaccuucuauugcg
novel21	uagcagugcuuuugaucgaguu	novel63	gccgauccgucgccgucgag
novel22	acagcacgaggcaggcggugcg	novel64	acacgugcugccgacuagugg
novel23	uagcagugcuuuugaucgaguu	novel65	gaugucuggaaccuucuauugcg
novel24	ccgucagugcauugguaggucg	novel66	gccuuccaucuccaaguu
novel24	ccaggucaugaaggcgagagag	novel67	uauaggagagagaaaaagacuga
novel26	gaacaaacaaaguaaguacaug	novel68	ugcucaucaaagcuggcugugaua
novel27	acgaaucaacgguuccccg	novel69	aagccuacuccuaguauaaua
novel28	gcauugagaucgcgaaucga	novel70	aauagcuauuacauucccaguga
novel29	ucggccucugaaccguaauua	novel71	uagccgcugucacacgcacaag
novel30	acuuccacuacgucacaucaca	novel72	auuuugcgaucgccaaaacgag
novel31	gccacgccuccgccucgcacu	novel73	acaucacagaucuguuauua
novel32	ucggccucugaaccguaauua	novel74	acaagaagcuucaaacacacag
novel33	aguuuacagguucccuca	novel75	auagcuauuacauacccaguga
Novel34	acgugcguauugaagaucua	Novel76	cguugcacuugcauuaauuauucu
Novel35	cccagaauuaccagcugauauuu	Novel77	agcaaagcccgaaugcgaaacu
Novel36	gauguccguagcccggggauuc	Novel78	uucaaaccucaugucucaag
Novel37	gcgugagcaccgcgugacguca	Novel79	uagguuagaauucuagcu
Novel38	aagaauccguaauccacuuauu	Novel80	gaaugcuugguuuuucuauccuu
Novel39	gcgccccuuggcagcuacagggu	Novel81	acaugguacaaguuugcagaucu
Novel40	gccggcgaacgcggguccg	Novel82	acaugguacaaguuugcagaucu
novel41	gccggcgaacgcggguccg	Novel83	aauauggucucauuacuacguaa
novel42	uagcgauucuaaacgauaagau		
